# Nanoparticles Influence Lytic Phage T4-like Performance In Vitro

**DOI:** 10.3390/ijms23137179

**Published:** 2022-06-28

**Authors:** Xymena Stachurska, Krzysztof Cendrowski, Kamila Pachnowska, Agnieszka Piegat, Ewa Mijowska, Paweł Nawrotek

**Affiliations:** 1 Department of Microbiology and Biotechnology, Faculty of Biotechnology and Animal Husbandry, West Pomeranian University of Technology in Szczecin, Piastów Avenue 45, 70-311 Szczecin, Poland; pawel.nawrotek@zut.edu.pl; 2 Faculty of Civil and Environmental Engineering, West Pomeranian University of Technology in Szczecin, Piastów Avenue 50a, 70-311 Szczecin, Poland; krzysztof.cendrowski@zut.edu.pl; 3 Department of Horticulture, Faculty of Environmental Management and Agriculture, West Pomeranian University of Technology in Szczecin, Juliusza Słowackiego 17, 71-434 Szczecin, Poland; kamila.pachnowska@zut.edu.pl; 4 Department of Nanomaterials Physicochemistry, Faculty of Chemical Technology and Engineering, West Pomeranian University of Technology in Szczecin, Piastów Avenue 45, 70-311 Szczecin, Poland; ewa.mijowska@zut.edu.pl; 5 Department of Polymer and Biomaterials Science, Faculty of Chemical Technology and Engineering, West Pomeranian University of Technology in Szczecin, Piastów Avenue 42, 71-065 Szczecin, Poland; agnieszka.piegat@zut.edu.pl

**Keywords:** nanoparticles, lytic bacteriophage, interaction, physical attachment, zeta potential

## Abstract

Little is known about interactions of non-filamentous, complex-structured lytic phages and free, non-ordered nanoparticles. Emerging questions about their possible bio-sanitization co-applications or predictions of possible contact effects in the environment require testing. Therefore, we revealed the influence of various nanoparticles (NPs; SiO_2_, TiO_2_-SiO_2_, TiO_2_, Fe_3_O_4_, Fe_3_O_4_-SiO_2_ and SiO_2_-Fe_3_O_4_-TiO_2_) on a T4-like phage. In great detail, we investigated phage plaque-forming ability, phage lytic performance, phage progeny burst times and titers by the eclipse phase determinations. Additionally, it was proved that TEM micrographs and results of NP zeta potentials (ZP) were crucial to explain the obtained microbiological data. We propose that the mere presence of the nanoparticle charge is not sufficient for the phage to attach specifically to the NPs, consequently influencing the phage performance. The zeta potential values in the NPs are of the greatest influence. The threshold values were established at ZP < −35 (mV) for phage tail binding, and ZP > 35 (mV) for phage head binding. When NPs do not meet these requirements, phage–nanoparticle physical interaction becomes nonspecific. We also showed that NPs altered the phage lytic activity, regardless of the used NP concentration. Most of the tested nanoparticles positively influenced the phage lytic performance, except for SiO_2_ and Fe_3_O_4_-SiO_2_, with a ZP lower than −35 (mV), binding with the phage infective part—the tail.

## 1. Introduction

Bacteriophages (phages), viruses specific to bacterial cells, are a powerful tool for building a variety of self-organizing nanostructures that can be used in the diagnosis and treatment of many diseases [1]. Interest in phages grew rapidly in the face of the ineffectiveness of routinely used therapeutic methods, although their use has not yet been widely adopted by modern medicine, with the exception of Georgia, Russia and Poland, where therapeutic phages have been used for a long time [2]. They show a significant antibacterial potential and, as passable bacteriolytic units, they are used in the mono-phage or multi-phage (phage cocktails) therapy of bacterial diseases, including those caused by multi-drug-resistant strains [2,3,4]. Phages represent the most numerous (approx. 10^31^) and probably the oldest, the most genetically diverse and the fastest replicating (approx. 10^23^ infections/s. on the Earth scale) biological form [5,6]. The biochemical complexity of the molecules that make up the phage virion and its varied size, expressed on the nanometric scale, mean that bacteriophages can be treated as natural nanoparticles made of molecules with different charge and hydrophobicity, which can also interact with other materials, including nanomaterials [7,8,9].

Nanomaterials, in turn, are structures produced at the nano scale, at least one dimension of which ranges from 1 to 100 nm, and thanks to the favorable ratio of surface area to their volume, they can be more biologically active than macroparticles with the same chemical composition [10,11,12,13,14]. The sizes of nanomaterials, similar to subcellular elements, allow them to penetrate through natural barriers, such as biological membranes, so that, after being introduced into the body, they can reach the smallest vessels and any types of cells [10,11]. Currently, due to the large variety of nanomaterials, they are proposed for use in the diagnosis and treatment of diseases (fluorescent biological labels, drug and gene delivery, detection of pathogens and proteins, probing of DNA structure, tissue engineering, tumor destruction, separation and purification of biological molecules), manufacturing and materials, environment, electronics, energy harvesting, food and agriculture [15,16,17]. Above all, their high antimicrobial activity is emphasized, which is an alternative to the currently used methods of neutralizing pathogens [18,19].

The application potential of bacteriophages or nanomaterials is currently being intensively developed, and at the same time, attempts are being made to increase their action and significance by obtaining synergistic effects [20,21]. On the other hand, the creation of biohybrids composed of both inorganic nanoparticles and phage biomolecules, opens new potential applications in very different fields, including medicine or nanobiotechnology [22,23].

Prospectively, it is possible to develop specific strategies based on a combination of phage and nanomaterials, used not only for eradication but also for sanitization, biocontrol or bioconservation, in the case of most bacterial pathogens, including drug-resistant and biofilm-forming bacteria [24,25]. It is also desirable to have an enhanced bactericidal effect, most preferably synergy, as it is proven for some antibiotics and bacteriophages [3,26,27]. In the case of phage–antibiotic synergy (PAS), it was shown that antibiotic and phage resistance have a small probability of emergence at the same time and bacteria resistant to one agent will be susceptible to the other agent [28]. Moreover, selective pressure of bacteriophage promotes phage-resistant mutations in bacteria, although with a weakening of their fitness. Such fitness trade-offs include reduced virulence, resensitization to antibiotics and colonization defects [29,30,31,32,33]. Therefore, similar effects can be expected in the context of the simultaneous use of lytic bacteriophages and nanoparticles.

With the current state of the art, the topic of phage–nanoparticle interactions mostly focuses on designing novel, functional materials and composites, mainly bioactive surfaces and biotemplate designs [34]. However, not much is known about the contact effects of non-filamentous lytic phages (especially those of complex head–tail structure) and non-ordered nanoparticles, with particular emphasis on their interactions. For that reason, in this work, we assessed the potential in lytic phage T4-like and nanoparticle co-application, primarily with a focus on the potential influence of nanoparticles on phage lytic activity.

## 2. Materials and Methods

### 2.1. Bacterial Host and Bacteriophage

Lytic bacteriophage T4_5_ was used, as a model for T4-like phages. *Escherichia coli* K-12 C600 [35] was used as a host strain for the phage propagation [3]. Bacterium and phage were part of a collection from the Department of Microbiology and Biotechnology at the West Pomeranian University of Technology, Szczecin. Bacterium revival and phage propagation were conducted as described previously [3]. Briefly, after storing in the Microbank™ system at −20 °C, the bacterium was revived on Luria-Bertani (LB agar) (BioMaxima, Lublin, Poland) plates and incubated at 37 °C for 24 h. Afterwards, 50 mL of LB broth was inoculated with a single colony of *E. coli*, and incubated at 37 °C with shaking (160 rpm) in an orbital rotating shaker (Shaker-Incubator ES-20, BioSan, Józefów, Poland) to reach OD_600nm_ = 0.5 (exponential phase of growth and a titer of approx. 2 × 10^8^ CFU). Optical density values were measured using an Infinite 200 PRO NanoQuant microplate reader (Tecan, Männedorf, Switzerland). Afterwards, bacteriophage was added and samples were further incubated until the lysis occurred. For lysate purification, chloroform was added (10%, *v/v*) and samples were vortexed for 5 min, and then centrifuged (Eppendorf Centrifuge model 5810 R, Hamburg, Germany) at 5000 rpm for 15 min at 4 °C. The supernatant was stored at 4 °C for further use. Phage activity and titer was tested by a double-overlay agar plaque assay [36].

### 2.2. Chemical Reagents

Titanium dioxide particles were obtained from the AEROXIDE^®^ TiO_2_ P 25 (Evonik, Essen, Germany). The Fe_3_O_4_ particles used as a core for the nanostructures and as reference samples were purchased from Sigma Aldrich (637106) and were used as received. The nanomagnetite structure size was 50–100 nm (purity 97%, according to information provided by the supplier). Titanium (IV) butoxide (TBT) and hexadecyl (trimethyl)azanium bromide (CTAB) were purchased from Sigma Aldrich (MERCK, Darmstadt, Germany). Ethanol, propanol and 30% hydrochloric acid ammonia solution were bought from Chempur (Piekary Slaskie, Poland).

### 2.3. Nanoparticle Synthesis

#### 2.3.1. Synthesis of Silica Shell on Iron Oxide Nanoparticles (SiO_2_-Fe_3_O_4_) and Their Functionalization with Titanium Dioxide (SiO_2_-Fe_3_O_4_-TiO_2_)

In order to create a mesoporous shell on the surface of iron oxide particles (Fe_3_O_4_-SiO_2_), 2 g of Fe_3_O_4_ and 0.930 g of hexadecyl (trimethyl)azanium bromide (CTAB) were dispersed in 200 mL of ethanol (EtOH) mixed together with 300 mL of water and 2.5 mL of ammonia solution (NH_3_ × H_2_O). The dispersion of the iron oxide particles in ethanol–water solution was obtained by simultaneous stirring and sonication. Further, 1.5 mL of tetraethyl orthosilicate (TEOS) was added and kept stirring for 18 h at room temperature. The obtained particles were separated with a magnet and dried in air. In the last step, the nanostructures were annealed at 500 °C for 2 h to remove CTAB blocking the pores. The silica shell synthesis was reported previously [37].

Iron oxide particles with mesoporous silica shell (Fe_3_O_4_-SiO_2_) were functionalized with titanium dioxide according to previously described method [38,39]. Fe_3_O_4_-SiO_2_ structures after annealing were added to the concentrated TBT in order to fill pores with TiO_2_. The mixture of Fe_3_O_4_-SiO_2_ and TBT was then sonicated for 2 h in an ultrasonic washer at 50 °C. Then Fe_3_O_4_-SiO_2_ structures were separated from the concentrated TBT using magnet and washed with isopropanol. After washing with isopropanol, nanostructures were washed with ethanol to hydrolyze remaining TBT and dried in air at 60 °C. The dried material was heated in an inert atmosphere for 2 h at 400 °C.

#### 2.3.2. Synthesis of Silica Nanospheres (SiO_2_) and Functionalization with Titanium Dioxide

Mesoporous silica nanospheres were prepared according to the previously reported Stöber method [39,40]. Briefly, synthesis of mesoporous silica nanospheres was performed by mixing 50 mL ethanol (EtOH) and 2.5 mL of ammonia solution (NH_3_ × H_2_O) together in a glass-bottomed flask. Ethanol and ammonium solution were stirred using magnetic stirrer. After mixing them together, 1.5 mL of tetraethyl orthosilicate (TEOS) was added to the flask and stirred for 24 h at room temperature. The obtained silica particles were obtained by evaporation solvents.

In the next step, to create a mesoporous external layer on the as-produced silica core particles (SiO_2_), mixture of 200 mg of silica spheres, 160 mg CTAB; 0.48 mL NH_3_ × H_2_O; 100 mL EtOH; and 120 mL H_2_O was prepared [40]. In order to homogeneously disperse the solution, obtained mixture was simultaneously stirred and sonicated. After obtaining a homogeneous suspension, 0.28 mL TEOS was added and the mixture was stirred for a further 24 h. The suspension was then evaporated and obtained product was annealed in air at 500 °C for 2 h. 

The modification of mesoporous channels in silica nanospheres with titanium dioxide (TiO_2_-SiO_2_) was made according to the previous procedure [38,39]. As such, 100 mg of silica core–shell structures was added to the 3 mL of concentrated TBT. Then, the suspension was sonicated for 3 h in anhydrous conditions at 50 °C. The suspension was then diluted with propanol and centrifuged to separate the excess of titanium dioxide precursor. In order to remove the excess of TBT, the sample was washed several times with propanol. After the purification step, the nanostructures filled with TBT were treated with ethanol to hydrolyze the precursor to the titanium dioxide. Finally, the sample was evaporated and heated in an air flow at 400 °C for 2 h to transform the titanium dioxide into its anatase phase.

### 2.4. Nanoparticle Microscopic Analysis, Zeta Potentials and Stock Suspension Preparation

Visualization of nanomaterials was performed using a transmission electron microscope (TEM; Tecnai G2 F20 S-TWIN; FEI, Hillsboro, OR, USA) equipped with a high-angle, annular, dark-field HAADF detector (STEM) and with energy-dispersive X-ray spectroscopy (EDS). In order to characterize the crystal structure of the samples, X-ray powder diffractometer (XRD, Aeris, Malvern Panalytical) with Cu-Kα radiation (λ = 1.544 Å) and an accelerating voltage of 40 kV was used.

For zeta potential analysis Zetasizer Nano ZS (Malvern Instruments, Malvern, UK) with a 633 nm red laser was used. All water-based dispersions of nanoparticles were sonicated for 30 min, directly before measurements in ultrasonic bath. For the analysis, the nanoparticle sample of the desired concentration (5 mg/mL) was flushed through a folded capillary cell and the measurement was carried out if there were no visible air bubble inclusions present. The cell was placed into the Zetasizer and equilibrated at 20 °C (close to the average temperature in the laboratory) for 2 min, of which there were three replications.

For stock suspension preparation, nanoparticles were placed in the glass tubes and sterile deionized water was added to reach a concentration of 10 mg/100 µL in each tube. Samples were thoroughly vortexed for 10 min and then sonified in the ultrasonic cleaner (2 × 30 min) by submerging the tubes to ensure particle dispersion.

### 2.5. Coincubation Assay for Plaque-Forming Ability Determination

In order to test phage T4_5_ plaque-forming ability in the presence of nanoparticles, coincubation assay was carried out. Briefly, 1980 µL of TM buffer (50 mM Tris-HCl, 10 mM MgSO_4_ at pH 7.5) and 20 µL of phage lysate (1.2 × 10^14^ PFU/mL) were added into wells of 12-well flat-bottom polystyrene plates. Then, nanoparticles were added to reach final concentrations of 0.5, 0.1 and 0.05 mg/mL. TM buffer with phage lysate and nanoparticle-free deionized water was used as a control. Samples were incubated at room temperature (22 °C) and 160 rpm in an orbital rotating shaker. Then, samples were collected immediately and after every 12 h, centrifuged at 5000 rpm for 2 min at 4 °C, and the supernatant was spotted on the double-layer agar plates. The experiment was conducted in triplicate.

### 2.6. Bacteriophage Lysis of Liquid Culture

For the host culture lysis studies in the presence of nanoparticles, 100 µL of an overnight culture of bacteria was refreshed in 2 mL of LB medium (BioMaxima, Lublin, Poland) in 12-well flat-bottom polystyrene plates and allowed to grow at 37 °C with shaking (160 rpm) until reaching OD_600nm_ = 0.2. Then each culture was infected with the phage at MOI (multiplicity of infection) = 0.1 and nanoparticles were added to reach final concentrations of 0.5, 0.1 and 0.05 mg/mL. The OD_600nm_ measurements of the samples were made every 30 min by collecting 100 µL of the sample supernatant into the 96-well flat-bottom polystyrene plates, in order to avoid false measurements caused by nanoparticles. LB medium was used as the blank sample, bacterial culture with phage-free buffer and nanoparticle-free deionized water was used as *E. coli* growth control and bacterial culture with phage lysate and nanoparticle-free deionized water was used as lysis control. The experiment was technically repeated three times, conducted in triplicate.

### 2.7. Determination of Phage Eclipse Period

In order to assess the time between phage infection and the appearance of phage progeny within the cell (intracellular phage/total phage) in the presence of nanoparticles, phage eclipse periods were estimated. As such, 10 mL of LB medium was inoculated with 100 µL of an overnight culture of *E. coli*. The culture was grown until OD_600nm_ = 0.5 and then centrifuged (3500 rpm for 5 min at 4 °C) and the pellet was suspended in 5 mL of fresh LB medium. At this point, the culture was infected with the phage at MOI = 0.01 and incubated with shaking (160 rpm) at 37 °C for 2 min. Then, 100 μL of infected cells was transferred to 5 mL of fresh LB broth in 12-well flat-bottom polystyrene plates and nanoparticles were added to reach final concentrations of 0.5, 0.1 and 0.05 mg/mL (time 0) and incubated at 37 °C with shaking (160 rpm). Samples were collected every 2 min, treated with chloroform (10%, *v*/*v*), vortexed for 1 min and then centrifuged at 5000 rpm for 5 min. The number of PFU per mL was estimated by titration using the double-layer agar plate method. The experiment was conducted in triplicate.

### 2.8. Microscopic Visualization of Phage–Nanoparticle Interactions

For phage purification, polyethylene glycol (PEG) 8000 (Pol-Aura, Dywity, Poland) with NaCl (Pol-Aura, Dywity, Poland) (final concentration 10% *w*/*v* PEG, 0.5 M NaCl) was added to the phage lysate and mixed thoroughly by inversion. The mixture was stored overnight at 4 °C, to allow the phage particles to form a precipitate. The precipitate was collected by centrifugation at 5000 rpm for 1.5 h at 4 °C. Supernatant was carefully discarded and the pellet was gently suspended in the TM buffer and left overnight at 4 °C. Then, PEG8000 was removed and phage particles were extracted by adding an equal volume of chloroform to the sample, vortexing for 20 min and centrifugating at 5000 rpm for 15 min at 4 °C. The upper phase containing phages was further purified by filtrating with a 0.22 µm PES filter. For phage morphology determination, phage particles were deposited on formvar/carbon-coated copper grids (400 mesh) and stained with UranyLess stain solution (Delta Microscopies, Mauressac, France) (the protocol available on http://uranyless.com, accessed on 1 October 2020). For phage–nanoparticle interaction visualization, purified phage T4_5_ was mixed with the highest nanoparticle concentrations (0.5 mg/mL), prior to deposition on grids and staining.

### 2.9. Statistical Analysis

One-way ANOVA was used to statistically analyze the results, along with Dunnett’s multiple comparisons test. Differences were considered significant at *p* ≤ 0.05. All statistical analyses were carried out using GraphPad Prism 8.01 (GraphPad Software, San Diego, CA, USA). All data are presented as mean with standard deviation (SD).

## 3. Results

### 3.1. Nanomaterials Characterization

**Figure 1:**
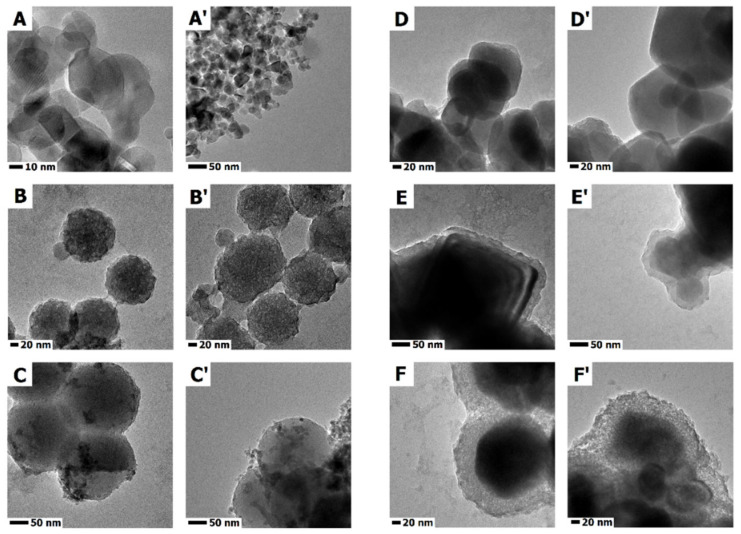
TEM images of titanium dioxide (**A**,**A’**); mesoporous silica spheres (**B**,**B’**); mesoporous silica spheres modified with titanium dioxide (**C**,**C’**); iron oxide particles (**D**,**D’**); covered with mesoporous silica shell before (**E**,**E’**) and after functionalization with titanium dioxide (**F**,**F’**).

TEM images of commercial titanium dioxide particles are presented in Figure 1A,A’. Titanium dioxide particles have a spherical-like shape with size in a range between 20 and 50 nm. According to information provided by the supplier, P25 has a surface area between 35 and 65 m^2^/g and chemical composition of anatase and rutile.

Detailed information on the iron oxide and iron oxide particles with silica shell is published in our previous work [37]. TEM images of the Fe_3_O_4_ and Fe_3_O_4_-SiO_2_ morphology show cube-like structures in a hexagonal shape, with particle size in a range of 50–100 nm for Fe_3_O_4_ (Figure 1D,D’) and 60–120 nm for Fe_3_O_4_-SiO_2_ (Figure 1E,E’). The Fe_3_O_4_ is composed from the magnetite phase, as are the Fe_3_O_4_-SiO_2_ particles [37].

The synthesized SiO_2_-Fe_3_O_4_-TiO_2_ is presented in Figure 1F,F’. SiO_2_-Fe_3_O_4_-TiO_2_ particles have similar shape and structure to SiO_2_-Fe_3_O_4_ particles, with solid, cubic core (from dense material) and mesoporous silica shell. The mesoporous silica shell of SiO_2_-Fe_3_O_4_-TiO_2_ is filled with titanium dioxide. On the surface of the silica shell of the SiO_2_-Fe_3_O_4_-TiO_2_, there are noticeable darker agglomerates of titanium dioxide. The XRD analysis presented in the Supplementary Material (Supplementary Material, Figure S1) proves that the structures are composed of iron oxide and titanium dioxide.

Detailed information on the SiO_2_ and TiO_2_-SiO_2_ chemical, physical and photocatalytic properties is presented in our previous publications [38,39]. Our work showed that the nanomaterials are characterized by high purity and high surface area. According to the TEM images, the synthesized materials have a spherical shape and are built from a solid core, with a mesoporous shell of SiO_2_ (Figure 1B,B’). TEM images presented in Figure 1C,C’ show that the mesoporous channels were filled with titanium dioxide. Additionally, titanium dioxide agglomerates (darker particles) can be noticed on the surface of the silica nanospheres. EDS mapping proved that the titanium dioxide is in the external mesoporous silica layer of TiO_2_-SiO_2_. The obtained materials are composed of silica, oxygen and titanium elements [40,41]. Titanium dioxide in mesopores is mostly in the anatase phase [41]. The synthesized photocatalyst shows high catalytic performance in the degradation of dyes [41], other organic pollutants [40] and shows antibacterial activity [42].

The Zeta potential analysis is presented in Table 1. From all of the studied materials, the highest maximal and minimal zeta potential charges had titanium dioxide (positive charge of 36 mV) and silica particles (negative charge −51 mV). The core–shell structure of silica and titanium dioxide TiO_2_-SiO_2_ also shows negative charge at −25 mV. The third important material used in the presented studies was iron oxide particles. These particles showed a slightly negative zeta potential charge of −1.1 mV. Due to the combination of iron oxide cores and silica shell, Fe_3_O_4_-SiO_2_ showed a highly negative charge at −37 mV. As presented in Table 1, titanium dioxide has the highest positive charge, but in combination with highly negative silica and almost passive iron oxide charge, the SiO_2_-Fe_3_O_4_-TiO_2_ particles showed negative charge at −19 mV.

### 3.2. Coincubation Assay

**Figure 2:**
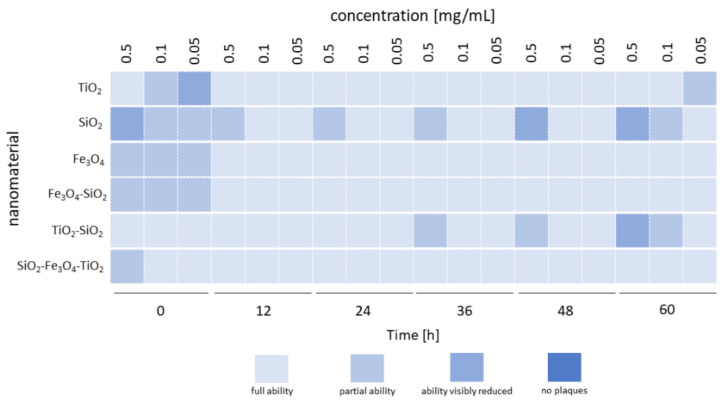
Phage plaque-forming ability after coincubation with selected nanoparticles.

The results of this experiment are shown in Figure 2. Phage T4_5_’s ability to form plaques after incubation with different nanoparticles was determined with comparison to the control (without the addition of nanoparticles). “Full ability” was assessed when spot-plaque morphology was the same as in the control spot-plaque (complete clearance of the bacterial lawn in the location of the spot); “partial ability” when in spot-plaque location, small, single phage plaques were observed; “ability visibly reduced” when in spot-plaque location, visible reduction of the small, single phage plaques was observed; “no plaques” when in spot-plaque location, no single phage plaques were observed. Detailed visualization of the results plate can be found in the Supplementary Material (Figure S2).

The most noticeable differences in the ability of the phage to form plaques occurred at time 0—immediately after adding the nanoparticles to the phage suspension (Figure 2). The highest concentration of nanoparticles (0.5 mg/mL) resulted in pronounced changes (“partial ability” or “ability visibly reduced”) in plaque visibility for most samples, but for titanium dioxide (TiO_2_), the lowest concentration (0.05 mg/mL) resulted in the most notable changes in the plaque-forming ability of the bacteriophage (“ability visibly reduced”). For samples with SiO_2_ nanoparticles, changes in the visibility of plaques were maintained throughout the incubation period (60 h), with the most abundant changes (“ability visibly reduced”) at 48 and 60 h of incubation, but only at the highest concentration of the nanoparticles (0.5 mg/mL). Changes were also recorded at 60 h of incubation, at a concentration of 0.1 mg/mL. In samples with TiO_2_-SiO_2_ nanoparticles, the reduction in plaque-forming ability was only observed at 36, 48 and 60 h of incubation at the highest nanoparticle concentration, and similarly to SiO_2_, at 60 h of incubation, at the concentration of 0.1 mg/mL (Figure 2).

### 3.3. Bacteriophage Lytic Performance

**Figure 3:**
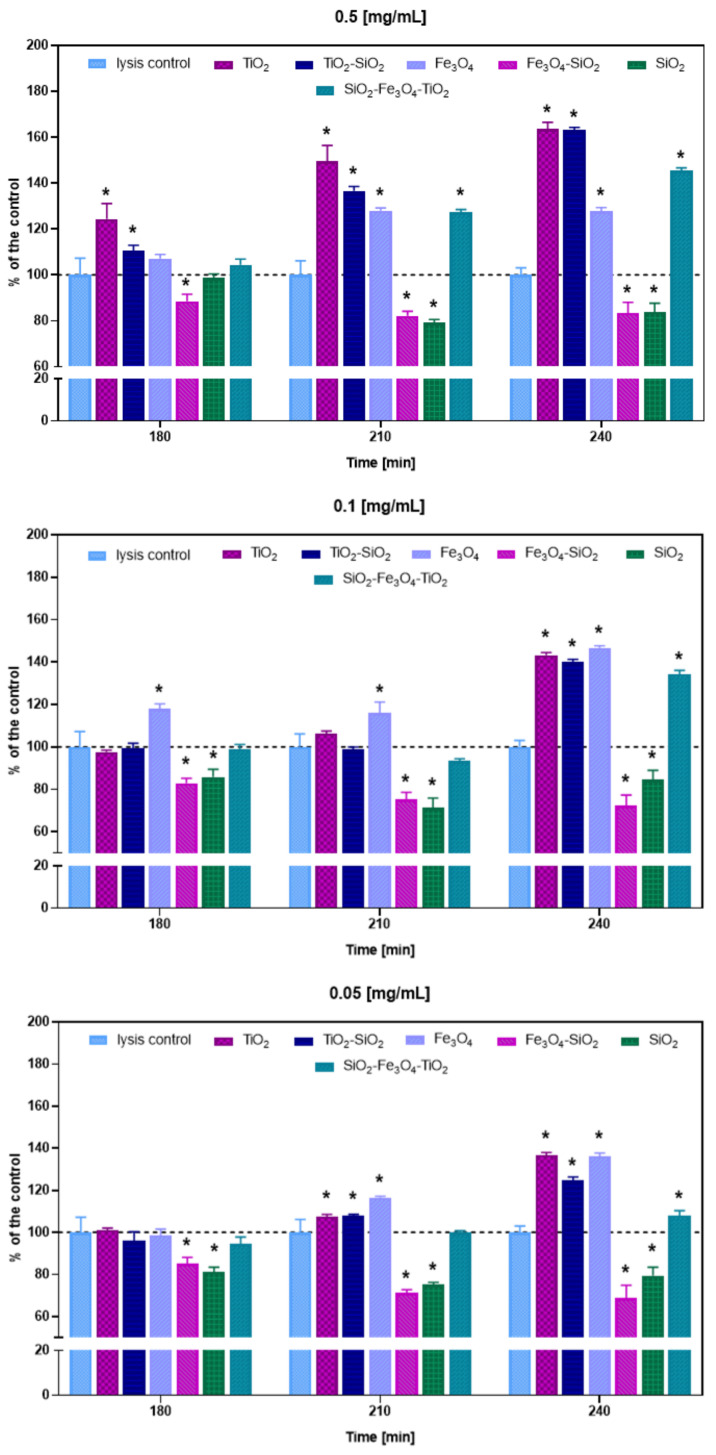
Area under the curve analysis of phage T4_5_ infection in the presence of nanoparticles at different concentrations (0.5, 0.1 and 0.05 mg/mL), at 180, 210 and 240 min of infection, as derived from lysis profile experiments equivalent to those presented in Supplementary Material (Figure S3). The smaller the area under a lysis profile curve, the greater the reductions in culture turbidity over time—better phage lysis performance. Percent above the dashed lines indicate higher optical density (OD) drops/greater reductions in culture turbidity compared to the control/better phage lysis. Percent below the dashed lines indicate lower OD drops/smaller reductions in culture turbidity compared to the control/weaker phage lysis. Error bars represent standard deviation between samples. Means sharing the star asterisk are significantly different from each other at *p* ≤ 0.05.

For the lysis ability test, bacteriophage T4_5_ performance against *E. coli* was tested in the presence of selected nanoparticles at different concentrations (0.5, 0.1 and 0.05 mg/mL), by the changes in optical density measures. Phage lytic performance was assessed by the analysis of areas under the lysis curves at 180, 210 and 240 min of incubation, when differences were most pronounced (Figure 3).

The quickest and most significant changes in phage lytic activity were observed at the highest (0.5 mg/mL) concentration of nanoparticles, and the highest percentage values occurred at 240 min of incubation. Compared to the lysis control, the presence of TiO_2_ resulted in increased lytic performance by 24%, 50% and 64% at 180, 210 and 240 min, respectively. Similarly, TiO_2_-SiO_2_ presence resulted in improved lytic performance by 10%, 37% and 63% at 180, 210 and 240 min, respectively. A significant improvement in T4_5_ lysis ability was also noted when Fe_3_O_4_ was added, but only at 210 and 240 min after phage infection, by 28%. Comparable results were observed for SiO_2_-Fe_3_O_4_-TiO_2_, where in the presence of the nanoparticles, phage lytic performance increased by 27% and 45% at 210 and 240 min, respectively. At a concentration of 0.5 mg/mL, reductions in phage lytic activity were also noted. This was the case with Fe_3_O_4_-SiO_2_ nanoparticles (decreased by 11%, 18%, 17% at 180, 210, 240 min, respectively) and SiO_2_ nanoparticles (decreased by 21% and 16% at 210 and 240 min, respectively).

At 0.1 mg/mL and 0.05 mg/mL, the greatest changes in percentage values occurred also at 240 min of incubation, but significant changes in reductive values were also detected earlier, at 180 and 210 min, whereas additive effects were present mostly at 210 and 240 min. At a concentration of 0.1 mg/mL, a significant improvement in T4_5_ lysis ability was noted for Fe_3_O_4_, which increased phage performance by 18%, 16% and 47% at 180, 210 and 240 min, respectively. Significant lysis improvement was also detected for TiO_2_ (43%), TiO_2_-SiO_2_ (40%) and SiO_2_-Fe_3_O_4_-TiO_2_ (34%) at 240 min after phage infection. Reductions in phage lytic activity were recorded for Fe_3_O_4_-SiO_2_ and SiO_2_, and were similar to those at 0.5 mg/mL (Fe_3_O_4_-SiO_2_: decreased by 17%, 25%, 28% at 180, 210, 240 min, respectively; SiO_2_: decreased by 14%, 29%, 15% at 180, 210, 240 min, respectively).

The reductions at a concentration of 0.05 mg/mL were also detected for the same nanoparticles and similar to those mentioned before (Fe_3_O_4_-SiO_2_: decreased by 15%, 29%, 31% at 180, 210, 240 min, respectively; SiO_2_: decreased by 19%, 25%, 21% at 180, 210, 240 min, respectively). A significant improvement in the phage lysis ability was detected only at 210 and 240 min of incubation. At 210 min, TiO_2_, TiO_2_-SiO_2_ and Fe_3_O_4_ presence resulted in the phage lytic boost by 8%, 8% and 17%, respectively, and at 240 min, these values further increased to 37%, 25% and 36%, respectively. At 240 min, an improvement in phage lytic performance was also noted for SiO_2_-Fe_3_O_4_-TiO_2_ (8%) (Figure 3).

### 3.4. Phage Eclipse Periods after Nanoparticle Exposure

**Figure 4:**
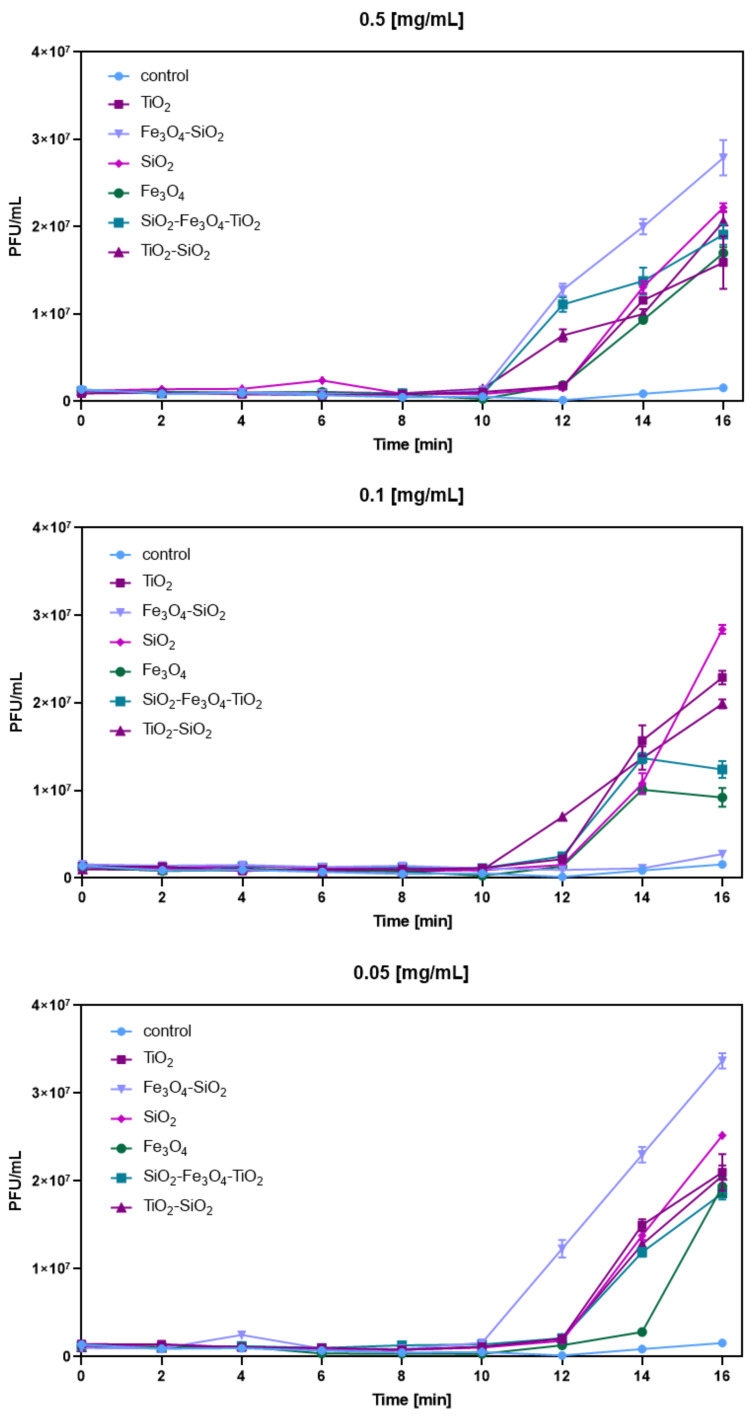
Bacteriophage T4_5_ eclipse periods in the presence of selected nanoparticles At concentrations of 0.5 mg/mL, 0.1 mg/mL and 0.05 mg/mL.

Phage T4_5_ eclipse periods were tested in order to assess the time between infection and the appearance of phage progeny within the cell (intracellular phage/total phage) in the presence of nanoparticles. Independently to the used nanoparticle concentration, the presence of most of them resulted in a quicker and greater production of phage particle progeny from bacterial cells, compared to the control, just after 12 min of incubation (Figure 4). The highest concentration (0.5 mg/mL) of all tested nanoparticles, namely Fe_3_O_4_-SiO_2_, SiO_2_-Fe_3_O_4_-TiO_2_, TiO_2_-SiO_2_, SiO_2_, TiO_2_ and Fe_3_O_4_, resulted in a significant increase in phage progeny numbers of 2.79 × 10^7^ (PFU/mL), 1.91 × 10^7^ (PFU/mL), 2.06 × 10^7^ (PFU/mL), 2.22 × 10^7^ (PFU/mL), 1.59 × 10^7^ (PFU/mL) and 1.7 × 10^7^ (PFU/mL) after 16 min of incubation compared to the control, respectively. At a concentration of 0.1 mg/mL, the presence of SiO_2_-Fe_3_O_4_-TiO_2_, TiO_2_-SiO_2_, SiO_2_, TiO_2_ and Fe_3_O_4_ resulted in the phage progeny numbers of 1.24 × 10^7^ (PFU/mL), 1.99 × 10^7^ (PFU/mL), 2.84 × 10^7^ (PFU/mL), 2.29 × 10^7^ (PFU/mL) and 0.92 × 10^7^ (PFU/mL) after 16 min of incubation compared to the control, respectively. That time, only Fe_3_O_4_-SiO_2_ presence did not alter the phage progeny numbers significantly (2.76 × 10^6^ PFU/mL; control—1.56 × 10^6^ PFU/mL). The lowest nanoparticle concentration (0.05 mg/mL) of Fe_3_O_4_-SiO_2_, SiO_2_-Fe_3_O_4_-TiO_2_, TiO_2_-SiO_2_, SiO_2_, TiO_2_ and Fe_3_O_4_ resulted in the phage progeny numbers of 3.37 × 10^7^ (PFU/mL), 1.86 × 10^7^ (PFU/mL), 2.06 × 10^7^ (PFU/mL), 2.52 × 10^7^ (PFU/mL), 2.1 × 10^7^ (PFU/mL) and 1.94 × 10^7^ (PFU/mL) after 16 min of incubation compared to the control, respectively (Figure 4). More detailed statistical analysis of the results of progeny phage numbers can be found in the Supplementary Material (Table S1).

### 3.5. Visualization of Phage–Nanoparticle Interaction

**Figure 5:**
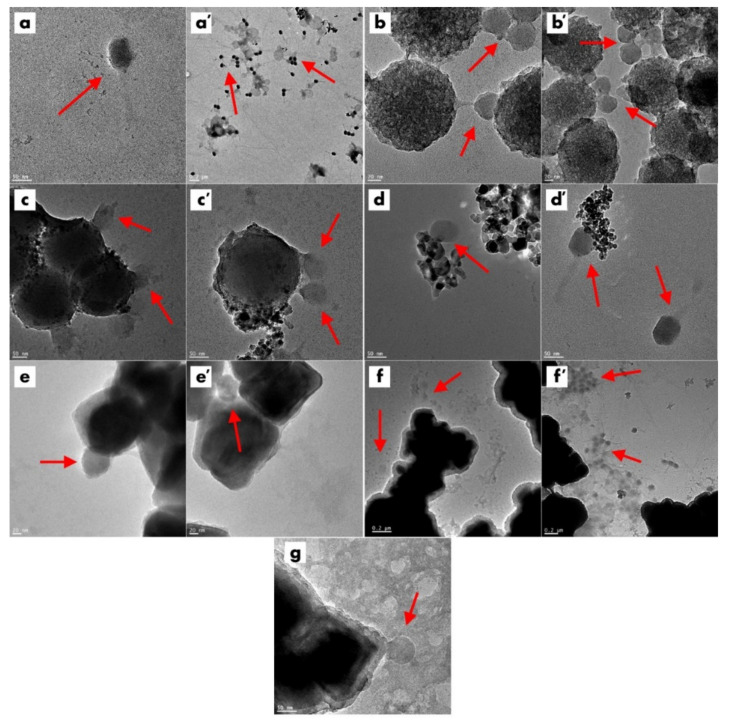
TEM micrographs of bacteriophage T4_5_ (**a**,**a’**) and of phage–nanoparticle interactions (**b**–**g**). Red arrows point to the phage particles. SiO_2_ and phage (**b**,**b’**), TiO_2_-SiO_2_ and phage (**c,c’**), TiO_2_ and phage (**d**,**d’**), Fe_3_O_4_ and phage (**e**,**e’**), SiO_2_-Fe_3_O_4_-TiO_2_ and phage (**f**,**f’**), Fe_3_O_4_-SiO_2_ and phage (**g**).

Coliphage T4_5_ was subjected to TEM to determine phage morphology and confirm phage classification that was assumed from our previous experiments. TEM images of the phage T4_5_ (Figure 5a,a’) resulted in classification of the virus into the *Caudovirales* order and the *Myoviridae* family, based on the typical phage morphological and infection features [43]. The images revealed that phages were in close proximity or in direct contact with nanoparticles. T4_5_ with SiO_2_ showed phages bound to silica spheres by their tails (Figure 5b,b’), in line with knowledge on the charge differences based on zeta potential (Table 1). Therefore, electrostatic interaction between phage and silica nanoparticles occurred. Negatively charged silica surfaces bind with positively charged phage tails. When silica spheres were coated with titanium nanoparticles (TiO_2_-SiO_2_), nonspecific phage binding happened, irrespective of the overall negative charge in the nanoparticles (Table 1)—phage particles were attached by their negatively charged heads (Figure 5c), as well as their positively charged tails (Figure 5c’). Phage T4_5_ in the presence of titanium dioxide attached to the positively charged TiO_2_ particles (Table 1) by the negatively charged head (Figure 5d,d’). When phage was in contact with Fe_3_O_4_ nanoparticles, the phage was again attached nonspecifically to the nanomaterial with close to neutral overall charge (Figure 5e,e’, Table 1). The presence of the negatively charged complex nanomaterial (SiO_2_-Fe_3_O_4_-TiO_2_) resulted in varied phage binding, with the predominance of phage flow in the direction of the nanoparticles (Figure 5f,f’). Fe_3_O_4_ nanoparticles covered by porous silica (Fe_3_O_4_-SiO_2_) with an overall negative charge were attached to the tail of the phage (Figure 5g). Additional images showing phage T4_5_ and nanoparticle interactions are presented in the Supplementary Material (Figure S4).

## 4. Discussion

With regard to phage–nanoparticle interactions, most of the work to date has focused on designing novel, functional materials or composites. The most common approach is using phages as scaffolds, nanocomposites or templates with nanoparticle addition. These are characterized as bioinorganic nanohybrids, composed of biological macromolecules and functional inorganic nanomaterials [44]. Since nanoscale inorganic materials are challenging in assembling into defined structures, the use of self-ordered biomolecules directly obtained from nature (nucleic acids, proteins, viruses) to organize nanomaterials into predesigned patterns is proposed. Bacteriophage–gold nanoparticle hybrids using phage M13 are one example [44]. The M13 phage is also mainly used for the phage display method—the process of identifying specific biological binding blocks using phage–bacteria biology through a directed evolution process—and the phage can recognize desired materials at the molecular level due to the recognition peptides [45]. An example of the use of phage display incorporating nanoparticles is the work of You et al. [46], in which two peptides were selected from a phage display peptide library by using ferromagnetic NPs as targets, since the selected peptides were verified to display strong binding affinity to Fe_3_O_4_ NPs. 

Another phage–nanoparticle combination approach is designing biosensors, which is often combined with phage display technology. Peng et al. [47] engineered phage M13 to display the receptor-binding protein that targets the desired bacteria and then thiolated the phages, which allowed the binding of gold nanoparticles that aggregated on the phages, resulting in a visible color change. Similarly, Souza et al. [48] fabricated “spontaneous, biologically active molecular networks” of filamentous phage and gold nanoparticles, working as biological sensors and cell-targeting agents, using peptide-displaying technology. With regard to designing bioactive surfaces with phages, physisorption by electrostatic forces and covalent bonding are most frequently used [34]. While covalent attachment offers a strong bond and phage detachment is not easy, it requires appropriate chemicals and rather complicated methodology [34,49]. A much simpler immobilization strategy uses the physical adsorption of the phage by electrostatic attraction between the phage and the surface [50].

However, little is known about interactions of non-filamentous lytic phages (especially of *Myoviridae* morphology) and free, non-ordered nanoparticles. This knowledge could be particularly useful for designing simple bio-sanitization solutions (co-application) or even prediction of phage and nanoparticle possible contact effects in the environment (potential influence of nanoparticles on phage lytic activity). Therefore, in our study, to understand the mechanisms of these phage–nanoparticle interactions, T4-like phage (T4_5_) was used as a model. T4-like phages (similarly as bacteriophage T4) have a complex head–tail structure that is characteristic of over 95% of all known bacteriophages. The *Myoviridae* phage consists of more than 40 different types of structural proteins which, simplifying, form a head and a contractile tail ending in a basal plate with six tail fibers. Almost half of these proteins are exposed to the external environment, with most of them being negatively charged at a pH of ~ 7 (their isoelectric point is pI < 7), except for the fiber ends, where the pI is > 7. Positively charged fibers are electrostatically attracted by bacteria that have a negatively charged surface [51]. Nanoparticles that were chosen for this study (SiO_2_, TiO_2_-SiO_2_, TiO_2_, Fe_3_O_4_, Fe_3_O_4_-SiO_2_ and SiO_2_-Fe_3_O_4_-TiO_2_) were characterized with different properties (Section 3.1, Nanomaterial Characterization) to ensure diversity in possible observed interactions. Nanomaterials were tested at the relatively low concentrations of 0.5, 0.1 and 0.05 mg/mL, since the concentrations of nanoparticles in the environment are unlikely to reach higher values [52]. A number of experiments were performed to determine: nanoparticle zeta potentials, phage plaque-forming ability after coincubation with nanoparticles and phage lytic performance in the presence of nanoparticles. Phage progeny boost times and titers were also determined by the eclipse phase determinations along with TEM micrographs of the interactions. Microscopic analysis of the tested samples showcased phage–nanoparticle physical attachments (Table 2).

In this study, nanoparticle zeta potential (ZP) values along with electron microscopy images were crucial to explain the rest of the results. Since the overall ZP value for T4 phage is close to neutral (−1.1 ± 0.49 mV) [53], the results were interpreted based on the distribution of phage charges (negatively charged phage head and positively charged tail) [54].

**Figure 6:**
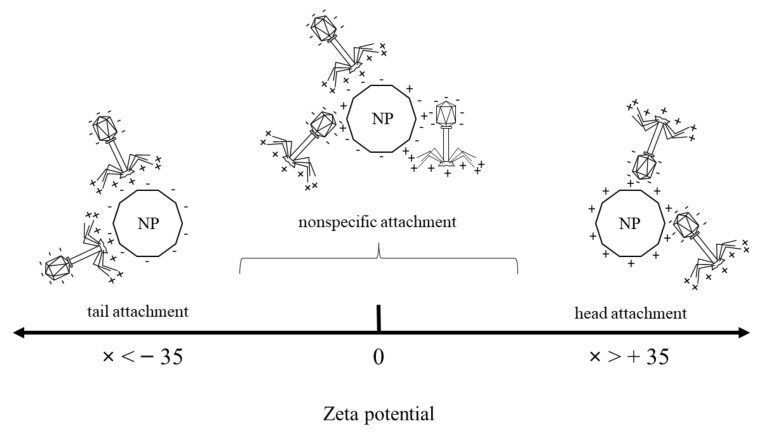
Proposed graphic explanation of the phenomenon of the electrostatic attachment of the phage T4-like and the tested nanoparticles. NP—nanoparticle.

Our results are interpreted with caution; however, it can be hypothesized that the mere presence of the nanoparticle charge, negative or positive, is not enough for the phage to attach specifically to the particle and consequently influence the phage performance. The zeta potential value of the nanoparticle is of the greatest influence—particles with a ZP lower than −35 [mV] bind effectively with positively charged phage tails, whereas particles with a ZP higher than 35 [mV] bind effectively with negatively charged phage heads (Figure 6). This was also additionally proven for CNT-TiO_2_ particles (Supplementary Material, Figure S5). When the particles do not meet these requirements, phage–nanoparticle physical interaction becomes nonspecific (Table 2).

These are in agreement with previous studies, where Cademartiri et al. [55] reported that tailed *Myoviridae* and *Siphoviridae* phages will effectively physisorb to porous silica spheres, rendered cationic or anionic by surface modification. Phage did not adsorb effectively to surfaces modified with neutral groups, and the number of infective phages bound to the silica was enhanced by the presence of ionic surfaces, with a greater surface charge correlating with greater concentration of adsorbed phages. The electrostatic attachment was also tested by Richter et al. [51], who engineered gold nanoparticles coated with a mixture of negatively charged 11-mercapto 1-undecanesulfonic acid (MUS) and hydrophobic 1-octanethiol (OT), which deactivated various types of *Escherichia coli*-selective phages: T1, T4 and T7. Moreover, the importance of the electrostatic forces was confirmed, since non-charged nanoparticles showed no effect in all analyzed cases. Thus, it was confirmed that the interactions between phages and nanoparticles can be predicted on the basis of the electrostatic forces between the averaged charges of phage proteins (represented by their pI) and the charges of nanoparticles [51]. We observed decreased phage lytic performance in the combination with SiO_2_ (ZP = −51 ± 0.3 [mV]) and Fe_3_O_4_-SiO_2_ (ZP = −37.6 ± 0.6 [mV]), which, as proposed by Khan et al. [56], is caused by the tail fiber target binding of the T4 phage; hence, the adsorption of the tail to a substrate (here, nanoparticles) limits the ability of T4 to bind targets (bacteria).

Tested nanoparticle concentrations had a minor influence on the phage–nanoparticle interactions, except for phage plaque-forming ability after coincubation with nanomaterials, and, only a slight influence on phage progeny boost time during the eclipse phase (±2 min) (Table 2). After coincubation, the phage was removed from the nanoparticle environment and, hence, the acquired phages could be the ones that: did not attach to the particles (small nanoparticles concentration/low availability), were left in the particle sediment after attachment, detached due to the small ZP value (t = 60 min) or did not have enough time to attach (t = 0 min). Moreover, it has been shown that even if the nanoparticles have a negative effect on phage lysis, when phage detaches, no permanent structural changes are incurred that produce inhibitory effects on infection after nanoparticles are removed [57]. Similarly, Khan et al. [56] noted that, contrary to predictions based on the charge differential for T4, the retention of activity was seen not only for phages adsorbed to cationic particles, but also for those bound to anionic spheres.

Similar to the present work, Gilcrease et al. [57] also tested whether bacteriophage lytic growth cycle is affected by the presence of nanoparticles, using silver nanoparticles with coating materials. One-step growth curves of bacteriophages showed that the presence of these nanoparticles resulted in 96% reductions in phages PFUs. However, when tailed *Siphoviridae* and *Myoviridae* phages were exposed to silver nanoparticles coated with poly-N-vinyl-2 pyrrolidone (PVP), final phage yield increased by 250%, compared with the same phage not incubated with nanoparticles. However, researchers suggest that this phenomenon is due to the coating itself, but the mechanism of phage stimulation by PVP was not revealed. In our results, we observed the cases of phage and negatively charged nanoparticles, in which eclipse periods showed quick phage progeny boost and titer, with a simultaneous decrease in phage lytic activity and phage tail binding (SiO_2_ and Fe_3_O_4_-SiO_2_). It can be hypothesized that after the first phage progeny burst, the phage particles are gradually captured over time by the negatively charged nanoparticles; hence, with each infective cycle, the amount of phage available to the bacteria decreases. In the cases where we noted increased lytic activity of the phage along with head binding to the positively charged nanoparticles (TiO_2_) or nonspecific phage binding (SiO_2_-TiO_2_, Fe_3_O_4_ and Fe_3_O_4_-SiO_2_-TiO_2_), it can be hypothesized that phage T4_5_ and nanoparticles worked in synergy. Similarly, You et al. [9] observed that 1 h prior exposure to silver and zinc oxide nanoparticles did not inactivate the MS2 phage at the highest nanoparticle concentrations tested (5 mg/L total Ag and 20 mg/L ZnO), but in a complex system where the E. coli was exposed to MS2 and nanoparticles simultaneously, the number of phages increased by 2–6 orders of magnitude.

A single T4-like phage virion is a colloidal particle, often having a dipole moment [7]. Therefore, it can be successfully bound to various nanomaterials with an appropriate (measurable) electric charge and increase or decrease the antimicrobial activity in the phage. For this reason, phages can be used not only in effective phage therapy, but also in biocontrol applications, e.g., in the food industry and agriculture, to protect dairy products, fruits, vegetables, meat and fish [7]. It should be noted, however, that the development of future disinfection solutions based on bacteriophages and nanoparticles may be hampered by the instability of phage and nanoparticle physical attachments, which could potentially be solved by the use of nanoparticles with, preferably, high zeta potentials.

## 5. Conclusions

In this paper, the interactions of six different nanoparticles and T4-like phages were revealed. Decreased phage lytic performance in the presence of nanoparticles having a ZP < −35 [mV] was observed. Particles with a low ZP cause the phage to attach with its tail fibers; hence, the adsorption of the phage tail to nanoparticle limits the ability of the phage to bind targets (bacteria). However, in some cases, retention of phage activity (plaque-forming ability) has been shown when the phage detached from the nanoparticle and was removed from the nanoparticle environment. No permanent changes were incurred that produced inhibitory effects on later phage infection. Increased phage lytic activity in the presence of nanoparticles was also noted. This was the case when the phage attached to the positively charged nanoparticles by the head, and when nonspecific phage binding took place, which could indicate antibacterial phage–nanoparticle synergy. However, generally, our results concerning nano–phage–bacteria mixtures are interpreted with caution, due to the probability of occurrence of some additional bacteria–nanoparticle interactions.

Overall, it was established that the mere presence of the nanoparticle charge is not sufficient for specific phage binding to a nanoparticle. The greatest influence in phage–nanoparticle interactions is the zeta potential (ZP) value of the nanoparticle. It was demonstrated that particles with a ZP lower than −35 (mV) bind effectively with positively charged phage tails, and particles with a ZP higher than 35 (mV) bind effectively with negatively charged phage heads. Particles which do not meet these requirements cause phage–nanoparticle physical interaction to become nonspecific. In consequence, interactions between phages and nanoparticles can be predicted on the basis of the particle charges expressed in ZP values.

## Figures and Tables

**Table 1:** Characteristics of the tested nanoparticles.

Material Type	Material Abbreviation	Zeta Potential [mV]
Silica nanospheres	SiO_2_	−51 ± 0.3
Meosporous silica nanospheres completed with titanium dioxide	TiO_2_-SiO_2_	−25 ± 0.4
Titanium dioxide	TiO_2_	36.5 ± 6
Iron oxide nanocubes	Fe_3_O_4_	−1.1 ± 1
Iron oxide nanocubes covered with silica shell	Fe_3_O_4_-SiO_2_	−37.6 ± 0.6
Iron oxide nanocubes covered with mesoporous silica shell completed with titanium dioxide	SiO_2_-Fe_3_O_4_-TiO_2_	−19.5 ± 0.2

**Table 2:** Key concentrated results of the present study, based on all of the obtained results.

Nanomaterial	Concentration [mg/mL]	Zeta Potential [mV]	Phage Plaque-Forming Ability at t = 0 h	Phage Plaque-Forming Ability at t = 60 h	Phage Lytic Performance after 240 min	Phage Progeny Boost Time	Phage Progeny Titer [PFU/mL]	Phage Attachment
SiO_2_	0.5	−51 ± 0.3	Visibly reduced	Visibly reduced	Decreased	From 12 min	2.22 × 10^7^	Tail
	0.1		Partial	Partial	Decreased	From 12 min	2.84 × 10^7^	
	0.05		Partial	Full	Decreased	From 12 min	2.52 × 10^7^	
SiO_2_-TiO_2_	0.5	−25 ± 0.4	Full	Visibly reduced	Increased	From 10 min	2.06 × 10^7^	Nonspecific
	0.1		Full	Partial	Increased	From 10 min	1.99 × 10^7^	
	0.05		Full	Full	Increased	From 12 min	2.06 × 10^7^	
TiO_2_	0.5	36.5 ± 6	Full	Full	Increased	From 12 min	1.59 × 10^7^	Head
	0.1		Partial	Full	Increased	From 12 min	2.29 × 10^7^	
	0.05		Visibly reduced	Partial	Increased	From 12 min	2.1 × 10^7^	
Fe_3_O_4_	0.5	−1.1 ± 1	Partial	Full	Increased	From 12 min	1.7 × 10^7^	Nonspecific
	0.1		Partial	Full	Increased	From 12 min	9.22 × 10^6^	
	0.05		Partial	Full	Increased	From 14 min	1.94 × 10^7^	
Fe_3_O_4_-SiO_2_	0.5	−37.6 ± 0.6	Partial	Full	Decreased	From 10 min	2.79 × 10^7^	Tail
	0.1		Partial	Full	Decreased	After 16 min	2.76 × 10^6^	
	0.05		Partial	Full	Decreased	From 10 min	3.37 × 10^7^	
Fe_3_O_4_-SiO_2_-TiO_2_	0.5	−19.5 ± 0.2	Partial	Full	Increased	From 10 min	1.91 × 10^7^	Nonspecific
	0.1		Full	Full	Increased	From 12 min	1.24 × 10^7^	
	0.05		Full	Full	Increased	From 12 min	1.86 × 10^7^	

## Data Availability

Not applicable.

## Supplementary Materials

The following supporting information can be downloaded at: www.mdpi.com/article/10.3390/ijms23137179/s1.
