# Supplementary material for: Nanoparticles Influence Lytic Phage T4-like Performance In Vitro
Source: Int J Mol Sci. 2022 Jun 28;23(13):7179. doi: 10.3390/ijms23137179 (PMC9266768; doi:10.3390/ijms23137179)
Supplement: Supplementary file 1 [file ijms-23-07179-s001.zip › ijms-1759657-supplementary.pdf]

Supplementary Materials

Nanoparticles influence lytic phage T4-like performance *in vitro*

Xymena Stachurska [1*], Krzysztof Cendrowski [2], Kamila Pachnowska [3,4], Agnieszka Piegat [5], Ewa Mijowska [4], Paweł Nawrotek [1]

[1] Department of Microbiology and Biotechnology, Faculty of Biotechnology and Animal Husbandry, West Pomeranian University of Technology in Szczecin, Piastów Avenue 45, 70-311 Szczecin, Poland; pawel.nawrotek@zut.edu.pl

[2] Faculty of Civil and Environmental Engineering, West Pomeranian University of Technology in Szczecin, Piastów Avenue 50a, 70-311 Szczecin, Poland; krzysztof.cendrowski@zut.edu.pl

[3] Department of Horticulture, Faculty of Environmental Management and Agriculture, West Pomeranian University of Technology in Szczecin, Juliusza Słowackiego 17, Szczecin 71-434, Poland; kamila.pachnowska@zut.edu.pl

[4] Department of Nanomaterials Physicochemistry, Faculty of Chemical Technology and Engineering, West Pomeranian University of Technology in Szczecin, Piastów Avenue 45, 70-311 Szczecin, Poland; ewa.mijowska@zut.edu.pl

[5] Department of Polymer and Biomaterials Science, Faculty of Chemical Technology and Engineering, West Pomeranian University of Technology in Szczecin, Piastów Avenue 42, 71-065 Szczecin, Poland; agnieszka.piegat@zut.edu.pl

* corresponding author: xymena.stachurska@zut.edu.pl

X-ray diffraction patterns of synthesized SiO_2-Fe_3O_4-TiO_2 particles

The X-ray diffraction patterns of synthesised SiO_2-Fe_3O_4-TiO_2 particles are presented in the Figure S1. From the presented pasterns characteristic peaks were assigned to the magnetite phase of iron oxide (JCPDS no. 65-3107), anatase (ICDD no. 03-065-5714) and rutile (ICDD no. 03-065-1118) phase of titanium dioxide. According to the TEM analysis, particles core is made from iron oxide and titanium dioxide is placed on the thin layer of mesoporous silica and small agglomerates form on the surface of the silica shell. Due to this, the prevailing intensity of the iron oxide over titanium dioxide peaks is in agreement with the TEM analysis of the SiO_2-Fe_3O_4-TiO_2 particles.

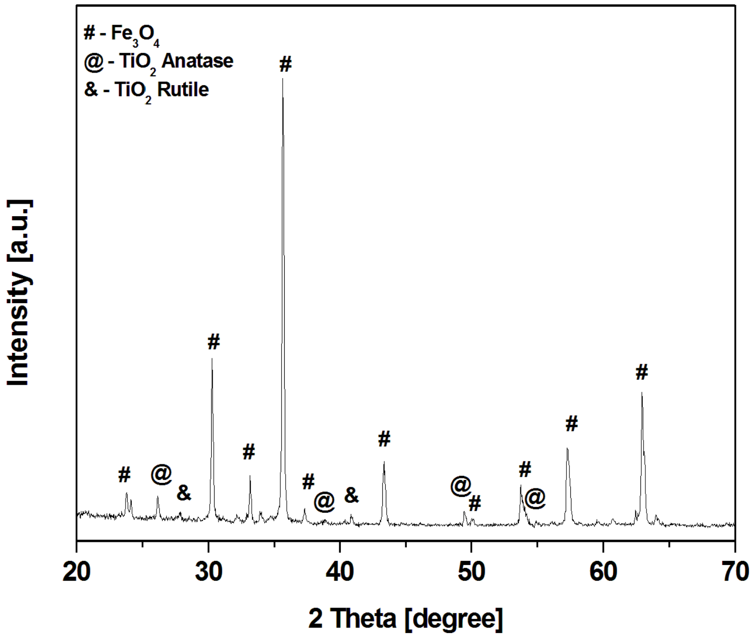

Figure S1. X-ray diffraction spectra of SiO_2-Fe_3O_4-TiO_2 particles with marked peaks corresponding to the Fe_3O_4 and TiO_2 phases.

Exemplary Petri plate from the coincubation test

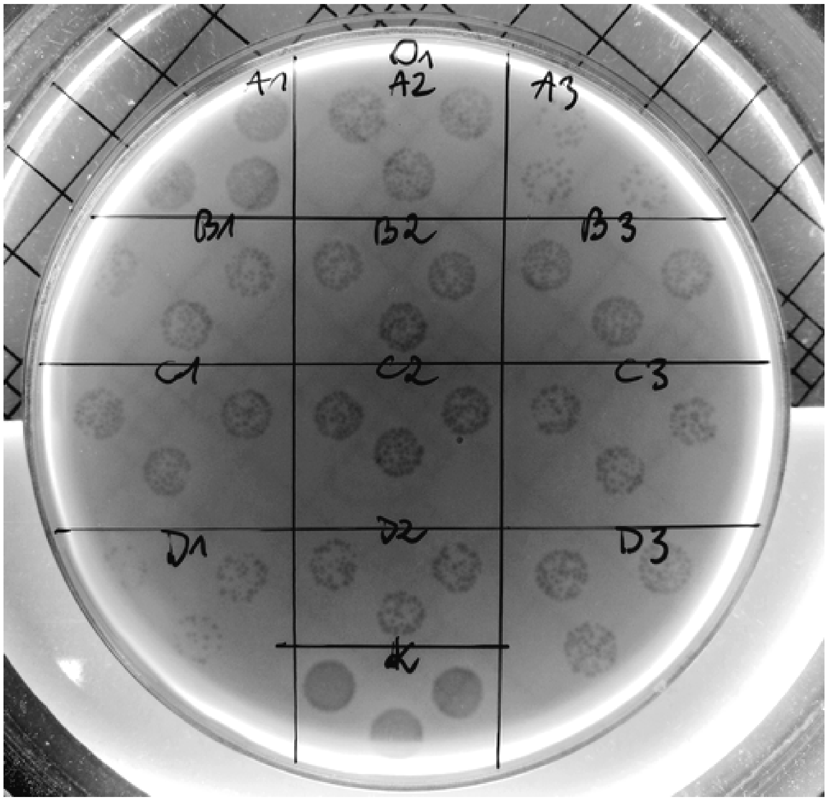

Figure S2. Detailed visualization of the results plate from the coincubation test.

E. coli lysis curves of the tested phage and nanoparticles

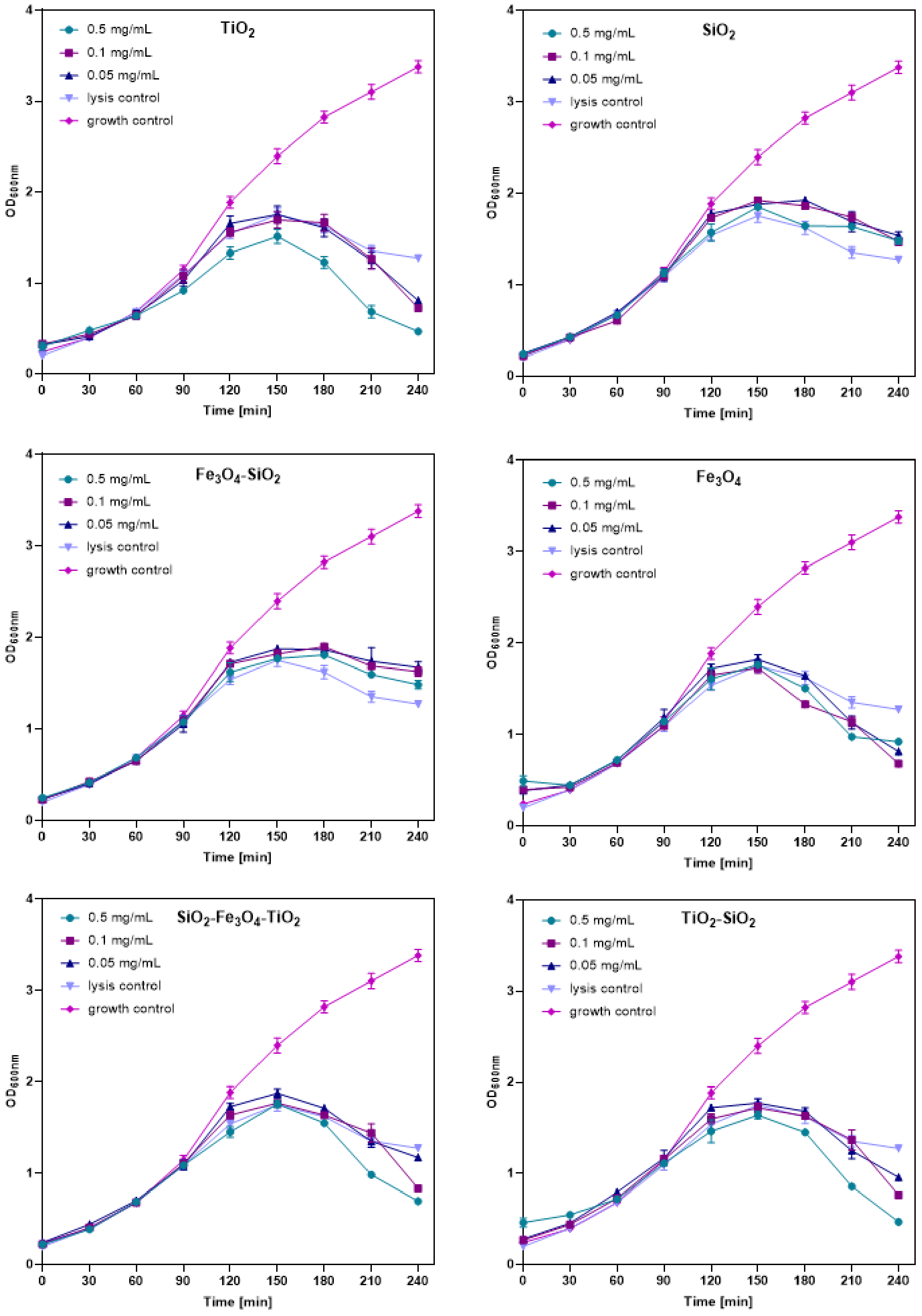

Figure S3. Lysis profile experiments of phage $T4_5$ infection in the presence of nanoparticles at different concentrations (0.5, 0.1 and 0.05 mg/mL).

Detailed statistical results of the phage eclipse periods test

Table S1. Dunnett's multiple comparisons test for significance interpretation of the results of progeny phage numbers, from the test of phage eclipse periods after nanoparticles exposure.

Dunnett's multiple comparisons test	Significant?	Summary	Adjusted P Value
	**0.5 mg/mL**		
**0 min**			
**control vs. TiO_2**	No	ns	0,8610
**control vs. Fe_3O_4-SiO_2**	No	ns	0,9998
**control vs. SiO_2**	No	ns	0,9995
**control vs. Fe_3O_4**	No	ns	0,9996
**control vs. SiO_2-Fe_3O_4-TiO_2**	No	ns	0,9977
**control vs. TiO_2-SiO_2**	No	ns	0,9975
**2 min**			
**control vs. TiO_2**	No	ns	0,9997
**control vs. Fe_3O_4-SiO_2**	No	ns	>0,9999
**control vs. SiO_2**	No	ns	0,8062
**control vs. Fe_3O_4**	No	ns	0,9975
**control vs. SiO_2-Fe_3O_4-TiO_2**	No	ns	>0,9999
**control vs. TiO_2-SiO_2**	No	ns	0,9997
**4 min**			
**control vs. TiO_2**	No	ns	0,9996
**control vs. Fe_3O_4-SiO_2**	No	ns	0,9996
**control vs. SiO_2**	No	ns	0,8279
**control vs. Fe_3O_4**	No	ns	0,9998
**control vs. SiO_2-Fe_3O_4-TiO_2**	No	ns	>0,9999
**control vs. TiO_2-SiO_2**	No	ns	>0,9999
**6 min**			
**control vs. TiO_2**	No	ns	>0,9999
**control vs. Fe_3O_4-SiO_2**	No	ns	0,9975
**control vs. SiO_2**	Yes	**	0,0021
**control vs. Fe_3O_4**	No	ns	0,9461
**control vs. SiO_2-Fe_3O_4-TiO_2**	No	ns	0,9959
**control vs. TiO_2-SiO_2**	No	ns	0,9591
**8 min**			
**control vs. TiO_2**	No	ns	0,9619
**control vs. Fe_3O_4-SiO_2**	No	ns	0,9959
**control vs. SiO_2**	No	ns	0,9309
**control vs. Fe_3O_4**	No	ns	0,9925
**control vs. SiO_2-Fe_3O_4-TiO_2**	No	ns	0,8646
**control vs. TiO_2-SiO_2**	No	ns	0,8646
**10 min**			
**control vs. TiO_2**	No	ns	0,7215
**control vs. Fe_3O_4-SiO_2**	No	ns	0,3828

**control vs. SiO_2**	No	ns	0,9724
**control vs. Fe_3O_4**	No	ns	0,9933
**control vs. SiO_2-Fe_3O_4-TiO_2**	No	ns	0,9882
**control vs. TiO_2-SiO_2**	No	ns	0,2317
**12 min**			
**control vs. TiO_2**	Yes	**	0,0040
**control vs. Fe_3O_4-SiO_2**	Yes	****	<0,0001
**control vs. SiO_2**	Yes	*	0,0157
**control vs. Fe_3O_4**	Yes	**	0,0018
**control vs. SiO_2-Fe_3O_4-TiO_2**	Yes	****	<0,0001
**control vs. TiO_2-SiO_2**	Yes	****	<0,0001
**14 min**			
**control vs. TiO_2**	Yes	****	<0,0001
**control vs. Fe_3O_4-SiO_2**	Yes	****	<0,0001
**control vs. SiO_2**	Yes	****	<0,0001
**control vs. Fe_3O_4**	Yes	****	<0,0001
**control vs. SiO_2-Fe_3O_4-TiO_2**	Yes	****	<0,0001
**control vs. TiO_2-SiO_2**	Yes	****	<0,0001
**16 min**			
**control vs. TiO_2**	Yes	****	<0,0001
**control vs. Fe_3O_4-SiO_2**	Yes	****	<0,0001
**control vs. SiO_2**	Yes	****	<0,0001
**control vs. Fe_3O_4**	Yes	****	<0,0001
**control vs. SiO_2-Fe_3O_4-TiO_2**	Yes	****	<0,0001
**control vs. TiO_2-SiO_2**	Yes	****	<0,0001
	**0.1 mg/mL**		
**0 min**			
**control vs. TiO_2**	No	ns	0,9998
**control vs. Fe_3O_4-SiO_2**	No	ns	0,9919
**control vs. SiO_2**	No	ns	0,9996
**control vs. Fe_3O_4**	No	ns	0,9996
**control vs. SiO_2-Fe_3O_4-TiO_2**	No	ns	0,9997
**control vs. TiO_2-SiO_2**	No	ns	0,7349
**2 min**			
**control vs. TiO_2**	No	ns	0,6667
**control vs. Fe_3O_4-SiO_2**	No	ns	0,4407
**control vs. SiO_2**	No	ns	0,9682
**control vs. Fe_3O_4**	No	ns	0,9996
**control vs. SiO_2-Fe_3O_4-TiO_2**	No	ns	0,8565
**control vs. TiO_2-SiO_2**	No	ns	>0,9999
**4 min**			
**control vs. TiO_2**	No	ns	0,9977
**control vs. Fe_3O_4-SiO_2**	No	ns	0,3916
**control vs. SiO_2**	No	ns	0,4533
**control vs. Fe_3O_4**	No	ns	0,9998
**control vs. SiO_2-Fe_3O_4-TiO_2**	No	ns	0,8112
**control vs. TiO_2-SiO_2**	No	ns	0,9958
**6 min**			
**control vs. TiO_2**	No	ns	0,8910
**control vs. Fe_3O_4-SiO_2**	No	ns	0,3627

**control vs. SiO_2**	No	ns	0,6458
**control vs. Fe_3O_4**	No	ns	0,9206
**control vs. SiO_2-Fe_3O_4-TiO_2**	No	ns	0,5078
**control vs. TiO_2-SiO_2**	No	ns	0,9998
**8 min**			
**control vs. TiO_2**	No	ns	0,4158
**control vs. Fe_3O_4-SiO_2**	Yes	*	0,0260
**control vs. SiO_2**	No	ns	0,4158
**control vs. Fe_3O_4**	No	ns	0,9059
**control vs. SiO_2-Fe_3O_4-TiO_2**	No	ns	0,1263
**control vs. TiO_2-SiO_2**	No	ns	0,8530
**10 min**			
**control vs. TiO_2**	No	ns	0,2635
**control vs. Fe_3O_4-SiO_2**	No	ns	0,3123
**control vs. SiO_2**	No	ns	0,7395
**control vs. Fe_3O_4**	No	ns	0,9469
**control vs. SiO_2-Fe_3O_4-TiO_2**	No	ns	0,2635
**control vs. TiO_2-SiO_2**	No	ns	0,7148
**12 min**			
**control vs. TiO_2**	Yes	****	<0,0001
**control vs. Fe_3O_4-SiO_2**	No	ns	0,0875
**control vs. SiO_2**	Yes	***	0,0002
**control vs. Fe_3O_4**	Yes	**	0,0020
**control vs. SiO_2-Fe_3O_4-TiO_2**	Yes	****	<0,0001
**control vs. TiO_2-SiO_2**	Yes	****	<0,0001
**14 min**			
**control vs. TiO_2**	Yes	****	<0,0001
**control vs. Fe_3O_4-SiO_2**	No	ns	0,9581
**control vs. SiO_2**	Yes	****	<0,0001
**control vs. Fe_3O_4**	Yes	****	<0,0001
**control vs. SiO_2-Fe_3O_4-TiO_2**	Yes	****	<0,0001
**control vs. TiO_2-SiO_2**	Yes	****	<0,0001
**16 min**			
**control vs. TiO_2**	Yes	****	<0,0001
**control vs. Fe_3O_4-SiO_2**	Yes	**	0,0016
**control vs. SiO_2**	Yes	****	<0,0001
**control vs. Fe_3O_4**	Yes	****	<0,0001
**control vs. SiO_2-Fe_3O_4-TiO_2**	Yes	****	<0,0001
**control vs. TiO_2-SiO_2**	Yes	****	<0,0001
	**0.05 mg/mL**		
**0 min**			
**control vs. TiO_2**	No	ns	0,9998
**control vs. Fe_3O_4-SiO_2**	No	ns	0,7528
**control vs. SiO_2**	No	ns	0,9935
**control vs. Fe_3O_4**	No	ns	0,9917
**control vs. SiO_2-Fe_3O_4-TiO_2**	No	ns	0,9969
**control vs. TiO_2-SiO_2**	No	ns	0,8973
**2 min**			
**control vs. TiO_2**	No	ns	0,4734
**control vs. Fe_3O_4-SiO_2**	No	ns	0,9998

**control vs. SiO_2**	No	ns	0,5806
**control vs. Fe_3O_4**	No	ns	0,9807
**control vs. SiO_2-Fe_3O_4-TiO_2**	No	ns	0,9935
**control vs. TiO_2-SiO_2**	No	ns	0,9994
**4 min**			
**control vs. TiO_2**	No	ns	0,9997
**control vs. Fe_3O_4-SiO_2**	Yes	****	<0,0001
**control vs. SiO_2**	No	ns	0,9976
**control vs. Fe_3O_4**	No	ns	0,9877
**control vs. SiO_2-Fe_3O_4-TiO_2**	No	ns	0,9667
**control vs. TiO_2-SiO_2**	No	ns	0,9788
**6 min**			
**control vs. TiO_2**	No	ns	0,8987
**control vs. Fe_3O_4-SiO_2**	No	ns	0,9797
**control vs. SiO_2**	No	ns	0,9976
**control vs. Fe_3O_4**	No	ns	0,8434
**control vs. SiO_2-Fe_3O_4-TiO_2**	No	ns	0,9246
**control vs. TiO_2-SiO_2**	No	ns	0,9976
**8 min**			
**control vs. TiO_2**	No	ns	0,8796
**control vs. Fe_3O_4-SiO_2**	No	ns	0,7104
**control vs. SiO_2**	No	ns	0,8796
**control vs. Fe_3O_4**	No	ns	0,9979
**control vs. SiO_2-Fe_3O_4-TiO_2**	No	ns	0,0762
**control vs. TiO_2-SiO_2**	No	ns	0,8796
**10 min**			
**control vs. TiO_2**	No	ns	0,3116
**control vs. Fe_3O_4-SiO_2**	Yes	*	0,0133
**control vs. SiO_2**	No	ns	0,5007
**control vs. Fe_3O_4**	No	ns	0,9870
**control vs. SiO_2-Fe_3O_4-TiO_2**	No	ns	0,0588
**control vs. TiO_2-SiO_2**	No	ns	0,3631
**12 min**			
**control vs. TiO_2**	Yes	****	<0,0001
**control vs. Fe_3O_4-SiO_2**	Yes	****	<0,0001
**control vs. SiO_2**	Yes	****	<0,0001
**control vs. Fe_3O_4**	Yes	**	0,0045
**control vs. SiO_2-Fe_3O_4-TiO_2**	Yes	****	<0,0001
**control vs. TiO_2-SiO_2**	Yes	****	<0,0001
**14 min**			
**control vs. TiO_2**	Yes	****	<0,0001
**control vs. Fe_3O_4-SiO_2**	Yes	****	<0,0001
**control vs. SiO_2**	Yes	****	<0,0001
**control vs. Fe_3O_4**	Yes	****	<0,0001
**control vs. SiO_2-Fe_3O_4-TiO_2**	Yes	****	<0,0001
**control vs. TiO_2-SiO_2**	Yes	****	<0,0001
**16 min**			
**control vs. TiO_2**	Yes	****	<0,0001
**control vs. Fe_3O_4-SiO_2**	Yes	****	<0,0001
**control vs. SiO_2**	Yes	****	<0,0001

**control vs. Fe_3O_4**	Yes	****	<0,0001
**control vs. SiO_2-Fe_3O_4-TiO_2**	Yes	****	<0,0001
**control vs. TiO_2-SiO_2**	Yes	****	<0,0001

ns – not significant

Additional TEM images of the phage and nanoparticles physical attachments.

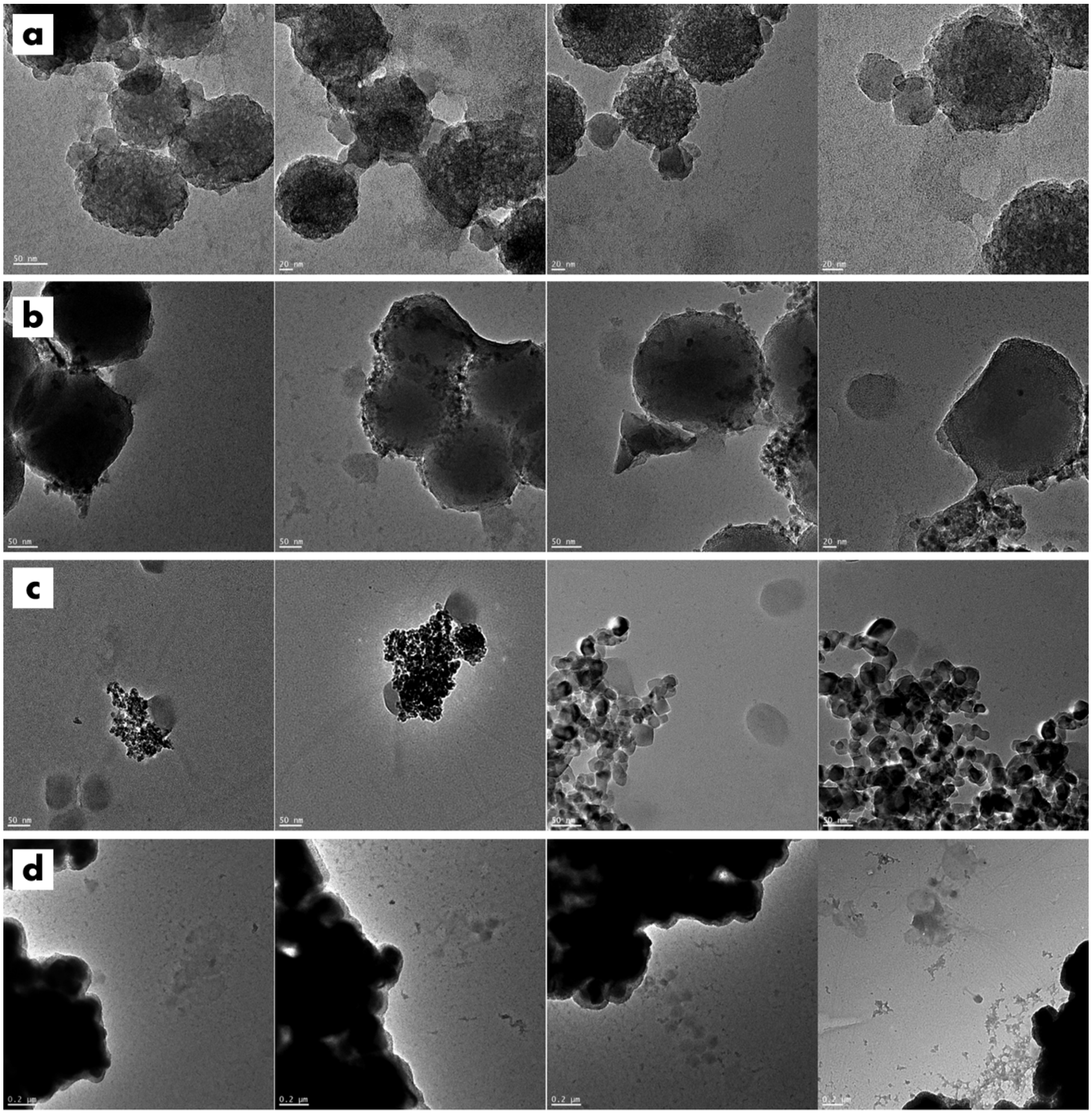

Figure S4. Additional TEM micrographs of phage-nanoparticles interactions (**a** – **d**). SiO_2 and phage (**a**), SiO_2-TiO_2 and phage (**b**), TiO_2 and phage (**c**), SiO_2-Fe_3O_4-TiO_2 and phage (**d**).

CNT-TiO_2 synthesis method and visualization of the interaction with the phage

For additional interactions visualization test, carbon nanotubes functionalized with titanium dioxide and phage was photographed. Firstly, the nanomaterial was synthesized. Carbon nanotubes

were purchased from Shenzhen Nanotech Port Co, (Shenzhen Nanotech Port Co, Shenzhen, China). Nanotubes modified with titanium dioxide (CNT-TiO_2) were prepared by using concentred titanium (IV) butoxide as a source of titanium dioxide. In order to obtain CNT-TiO_2, 20 mg of CNT previously functionalized with carboxyl groups was added to 1 mL concentrated solution of TBT. Then, the TBT and CNT were sonicated for three hours at the temperature of 50 °C. After sonication, the material was diluted with propanol and centrifuged (8000 rpm for 20 min) to remove the free titanium dioxide precursor. After washing the sample several times with propanol, the material was finally washed with ethanol. The TBT on the carbon nanotubes, exposed to the ethanol hydrolyzed to titanium dioxide. Finally, the sample was heated in airflow at 400 °C for 2 h. Then, according to the method described in the materials and methods section (2*.8. Visualization of phage-nanoparticles interactions*), nanoparticles and phages interactions were visualized (Figure S5). CNT-TiO_2 nanoparticles were also tested for their zeta potential values (40.3 ± 2 [mV]).

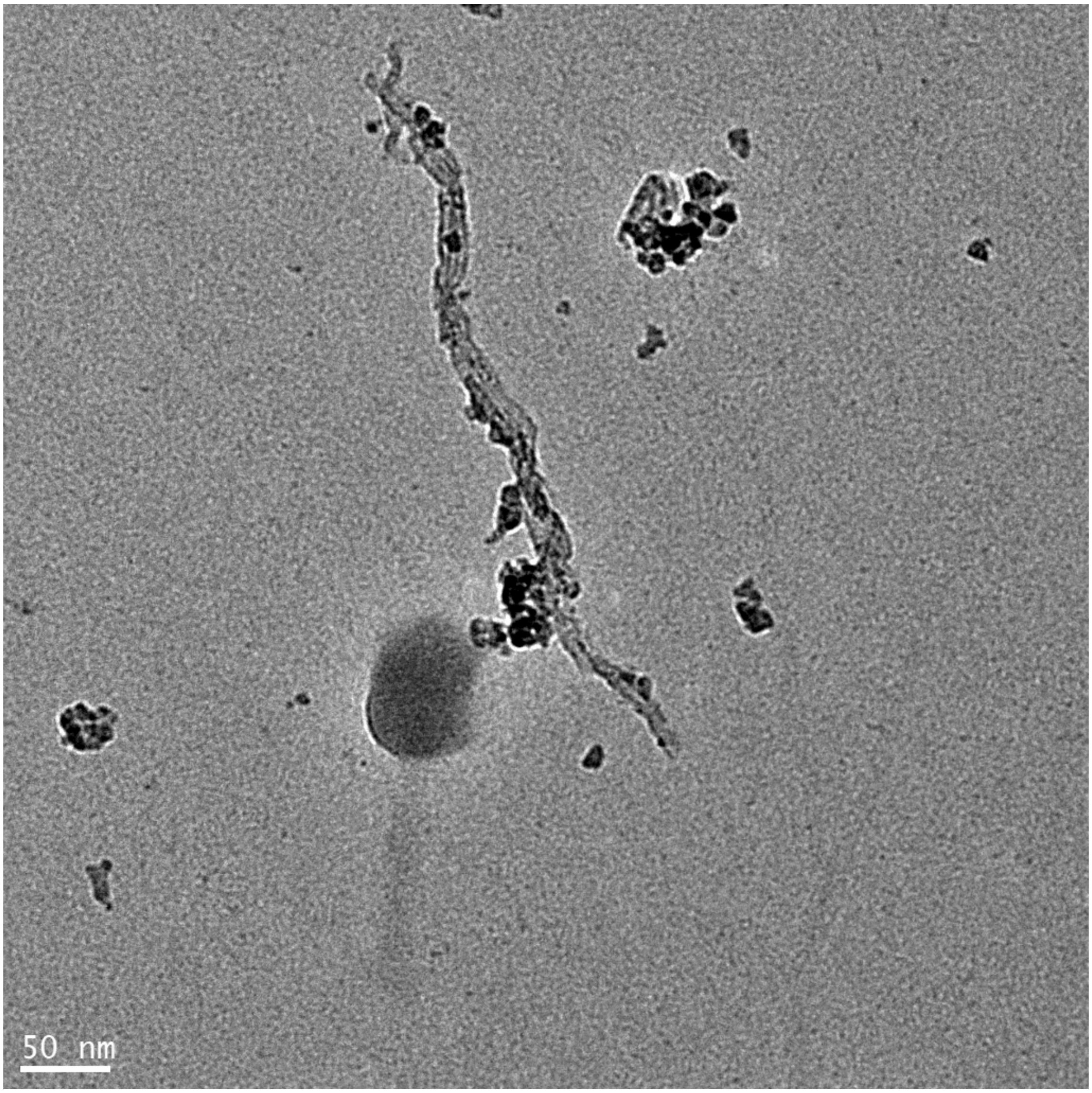

Figure S5. TEM micrograph of CNT-TiO_2 and $T4_5$ phage interaction.

Supplementary materials include: Results from the X-ray diffraction patterns of synthesized SiO_2-Fe_3O_4-TiO_2 particles (Figure S1: X-ray diffraction spectra of SiO_2-Fe_3O_4-TiO_2 particles with marked peaks corresponding to the Fe_3O_4 and TiO_2 phases); Exemplary Petri plate from the coincubation test (Figure S2: Detailed visualization of the results plate from the coincubation test); *E. coli* lysis curves of the tested phage and nanoparticles (Figure S3: Lysis profile experiments of phage $T4_5$ infection in the presence of nanoparticles at different concentrations (0.5, 0.1 and 0.05 mg/mL)); Detailed statistical results of the phage eclipse periods test (Table S1: Dunnett's multiple comparisons test for significance interpretation of the results of progeny phage numbers, from the test of phage eclipse periods after nanoparticles exposure); Additional TEM images of the phage and nanoparticle physical attachments (Figure S4: Additional TEM micrographs of phage–nanoparticle interactions (a–d). SiO_2 and phage (a), SiO_2-TiO_2 and phage (b), TiO_2 and phage (c), SiO_2-Fe_3O_4-TiO_2 and phage (d)); CNT-TiO_2 synthesis method and visualization of the interaction with the phage (Figure S5: TEM micrograph of CNT-TiO_2 and $T4_5$ phage interaction).
